# Anti-symmetric framework for balanced learning of protein–protein interactions

**DOI:** 10.1093/bioinformatics/btae603

**Published:** 2024-10-15

**Authors:** Tao Tang, Tianyang Li, Weizhuo Li, Xiaofeng Cao, Yuansheng Liu, Xiangxiang Zeng

**Affiliations:** School of Modern Posts, Nanjing University of Posts and Telecommunications, Nanjing 210023, China; School of Modern Posts, Nanjing University of Posts and Telecommunications, Nanjing 210023, China; School of Modern Posts, Nanjing University of Posts and Telecommunications, Nanjing 210023, China; School of Artificial Intelligence, Jilin University, Changchun 130012, China; College of Computer Science and Electronic Engineering, Hunan University, Changsha 410086, China; College of Computer Science and Electronic Engineering, Hunan University, Changsha 410086, China

## Abstract

**Motivation:**

Protein–protein interactions (PPIs) are essential for the regulation and facilitation of virtually all biological processes. Computational tools, particularly those based on deep learning, are preferred for the efficient prediction of PPIs. Despite recent progress, two challenges remain unresolved: (i) the imbalanced nature of PPI characteristics is often ignored and (ii) there exists a high computational cost associated with capturing long-range dependencies within protein data, typically exhibiting quadratic complexity relative to the length of the protein sequence.

**Result:**

Here, we propose an anti-symmetric graph learning model, BaPPI, for the balanced prediction of PPIs and extrapolation of the involved patterns in PPI network. In BaPPI, the contextualized information of protein data is efficiently handled by an attention-free mechanism formed by recurrent convolution operator. The anti-symmetric graph convolutional network is employed to model the uneven distribution within PPI networks, aiming to learn a more robust and balanced representation of the relationships between proteins. Ultimately, the model is updated using asymmetric loss. The experimental results on classical baseline datasets demonstrate that BaPPI outperforms four state-of-the-art PPI prediction methods. In terms of Micro-F1, BaPPI exceeds the second-best method by 6.5% on SHS27K and 5.3% on SHS148K. Further analysis of the generalization ability and patterns of predicted PPIs also demonstrates our model’s generalizability and robustness to the imbalanced nature of PPI datasets.

**Availability and implementation:**

The source code of this work is publicly available at https://github.com/ttan6729/BaPPI.

## 1 Introduction

Protein–protein interactions (PPIs) are arguably the most essential biological functions for maintaining biological activities (Chen *et al.* 2019, Lu *et al.* 2020). Identifying PPIs can provide in-depth information for elucidating the molecular mechanisms underlying biological processes and designing molecularly targeted therapies (Bakail and Ochsenbein 2016). The characteristics of PPIs naturally encapsulate an imbalanced distribution. For instance, the percentage of different functions can vary from 0.60% to 34.97% within the dataset of identified human interactome (Szklarczyk *et al.* 2023). Moreover, the graph representation of the PPI network also exhibits an imbalance in node degree. The inherent imbalance increases the cost of experimental verification and decreases the performance of computational prediction, posing significant challenges for studying PPI.

Over the past decade, due to the time-consuming and labor-intensive nature of wet experimental methods such as yeast two-hybrid (Ito *et al.* 2001) and tandem affinity purification (Bürckstümmer *et al.* 2006), many deep learning approaches have been proposed for systematic and efficient prediction of PPIs (Ahmed *et al.* 2018, Tang *et al.* 2023). In recent years, there has been a focus on constructing PPI graph to explore the structural information of each protein and the topological information within PPI networks. These advanced approaches can be categorized into two types based on the source of input data: sequence-based methods and structure-based methods.

Sequence-based methods have extensively been used in PPI prediction. In particular, graph neural network (GNN) and its variants demonstrate remarkable generalization capability to handle the relational information between proteins, making them preferred for PPI prediction, such as variational graph auto-encoder (Yang *et al.* 2020), graph isomorphism network (GIN) (Lv *et al.* 2021), graph attention network (GAN) (Kang *et al.* 2023), and GNN with self-supervised architecture (Zhao *et al.* 2023). These methods focus on the topological information in PPI network, and hence tend to overlook the network properties, especially node degrees. Besides GNN-based layers, other models have also been applied to multiple application scenarios (Chen *et al.* 2019, Yang *et al.* 2021), including binding affinity estimation and human-virus PPI prediction. From the perspective of sequential feature extraction, physicochemical properties serve as the primary input for deep learning models. Over the past years, advanced natural language processing (NLP) techniques are also preferred for the ability to extract local and global information simultaneously, including Transformer (Nambiar *et al.* 2020), Bidirectional Encoder Representations from Transformers (BERT) (Jha *et al.* 2023), and deep contextual language model (Rives *et al.* 2021).

There have also been many advances in extracting the spatial biological arrangements of residues from 2D or 3D structures (Yuan *et al.* 2022, Shen *et al.* 2024), contributing to both PPI prediction and protein representation learning (Bryant *et al.* 2022). The structural information is used either as a single source for prediction or incorporated with sequence data (Song *et al.* 2022). These methods normally apply structure-based approaches, often employing scoring functions or knowledge-based methods for PPI prediction (Soleymani *et al.* 2022). GNN is also extended to the representation of protein structure, forming a dual-view graph learning model (Gao *et al.* 2023, Rao *et al.* 2024) for PPI prediction that incorporates both bottom-inside-view (protein structure) and top-outside-view (PPI network structure). In addition to the standard GNN, heterogeneous GNNs are applied for handling the microenvironment of amino acid residues (Wu *et al.* 2024), and the 3D coordinate distances between carbon-alpha atoms in residues are utilized to define heterogeneous GNN.

Structure-based methods typically demonstrate better performance than sequence-based methods. Nevertheless, it requires atomic coordinates of proteins, making them unsuitable for protein datasets that contain only sequence data. Additionally, structure-based methods suffer from high computational costs for modeling structural information and hence are less efficient in training time (Wu *et al.* 2024). Moreover, both sequence-based and structure-based models often overlook the imbalanced nature of PPI datasets, such as the disparity in node degree and PPI characteristics, leading to bias in the classification of minority classes.

To address the aforementioned challenges, we propose a sequence-based method for the balanced learning of PPIs, namely *BaPPI*. The learning architecture is based on anti-symmetric operation and implicitly parameterized long convolutions. From the perspective of the PPI network, BaPPI models the uneven distribution of PPI networks using anti-symmetric graph convolutional network (GCN) to capture relational information in PPI network and prevent overfitting to certain characteristics. From the perspective of protein sequences, the long-range dependencies within encoded protein sequences are captured through a recurrent long convolution block, thus forming an efficient attention-free architecture for sequential data. Additionally, an asymmetric loss function is employed to adjust the penalties between errors of different protein types. We compare BaPPI with leading PPI prediction methods on the commonly used PPI baseline datasets SHS27K and SHS148K. The experimental results demonstrate the superiority of BaPPI in terms of both overall performance and the prediction of minority PPI types. The Micro-F1 of BaPPI surpasses the second-best method on four datasets by 6.5%, 5.3%, 8.1%, and 4.9%, respectively. In addition, the further ablation study shows that the integration of anti-symmetric operation and long convolution is particularly effective.

The primary contributions of our work consist of three main aspects: (i) achieving balanced learning of PPIs by using an anti-symmetric GCN, the regularization introduced by the anti-symmetric operation reduces the bias caused by the uneven distribution of node degree in PPI graph and imbalanced nature of PPI characteristics; (ii) the use of long convolution operations reduces the time complexity for capturing long-range dependencies of amino acid sequences, maintaining great efficiency in feature extraction; and (iii) integrating classical cross-entropy with an asymmetric focus to form an asymmetric loss function, thereby enhancing the generalization ability for identifying minority PPI types.

## 2 Materials and methods

The PPI prediction problem is formulated as follows: given a set of *n* proteins $P=\{p_{1},p_{2},\ldots ,p_{n}\}$, the PPI set is represented by $X=\{x_{ij}\},Y=\{y_{ij}\}$, where $x_{ij}=(p_{i},p_{j})$ denotes a pair of proteins and $y_{ij}$ denotes the corresponding PPI. The proteins and PPIs are considered as nodes and edges, respectively, to build PPI graph G=(P,X,Y). The objective is to learn a model $F:x\to \hat{y}$ from training set, *x* is the protein pair and $\hat{y}$ is the predicted PPI.

**Figure 1.**
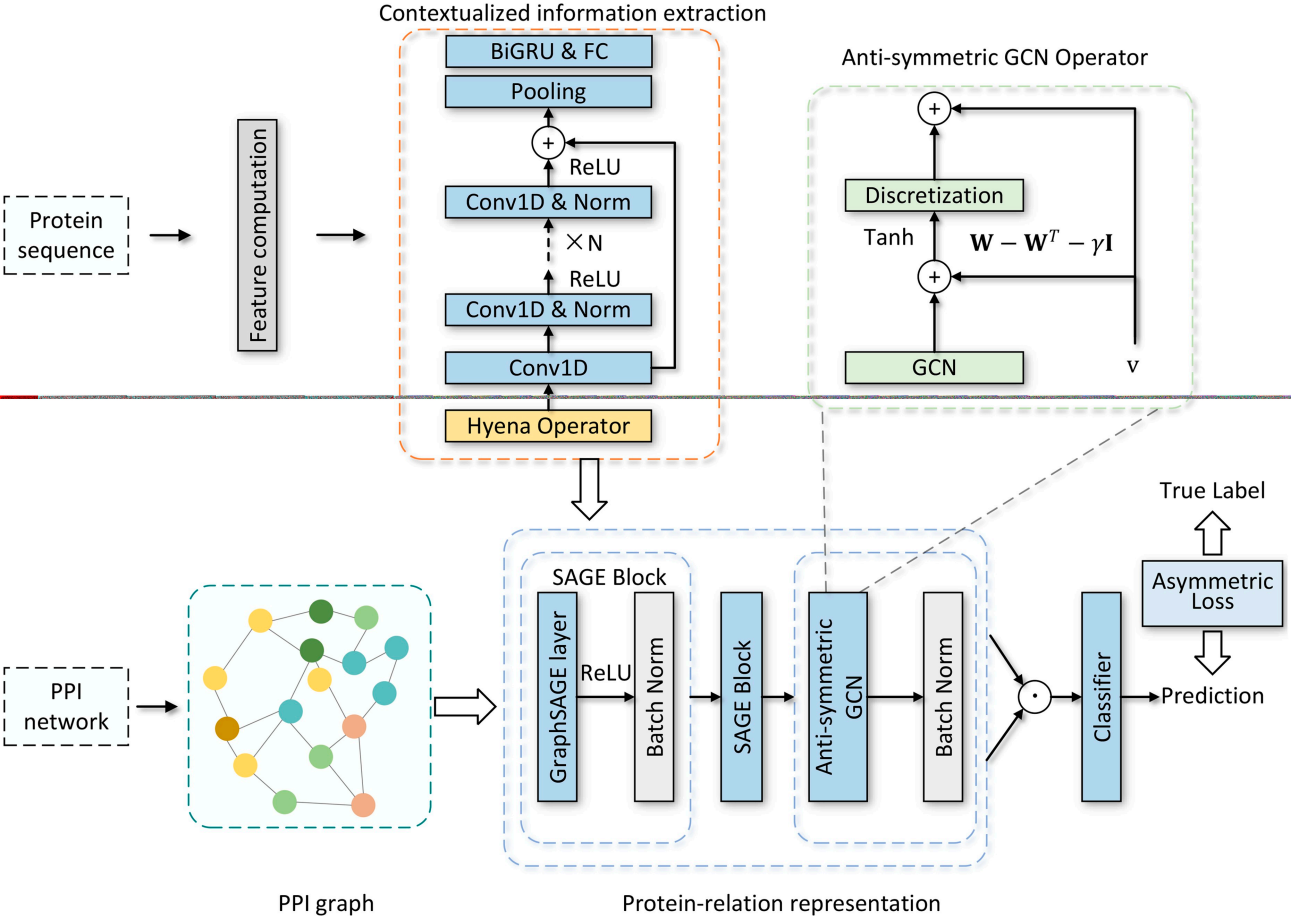
A schematic diagram of BaPPI consisting of five steps: (i) constructing the PPI graph from the PPI network data, which is used as the input adjacency matrix for the GNN, (ii) extracting contextualized sequence information via long convolution and residual 1D convolution, (iii) performing relational information extraction using GraphSaGE and an anti-symmetric GCN, (iv) computing the protein pair representation based on the neighborhood embedding of each protein and obtaining the PPI prediction with a classifier, and (v) updating the model weights by employing an asymmetric cross-entropy loss function.

Our proposed model, BaPPI, can be modularized into three main stages. The first stage extracts contextualized information using one-dimensional residual network (1D ResNet) and long convolution block. The second stage captures the long-range dependency between proteins using an antisymmetric GCN. The third stage performs PPI prediction via element-wise operation and a fully connected layer. The entire learning architecture is updated by asymmetric loss function. A schematic diagram of BaPPI is depicted in Fig. 1.

### 2.1 Long convolution for extracting contextualized information

**Figure 2.**
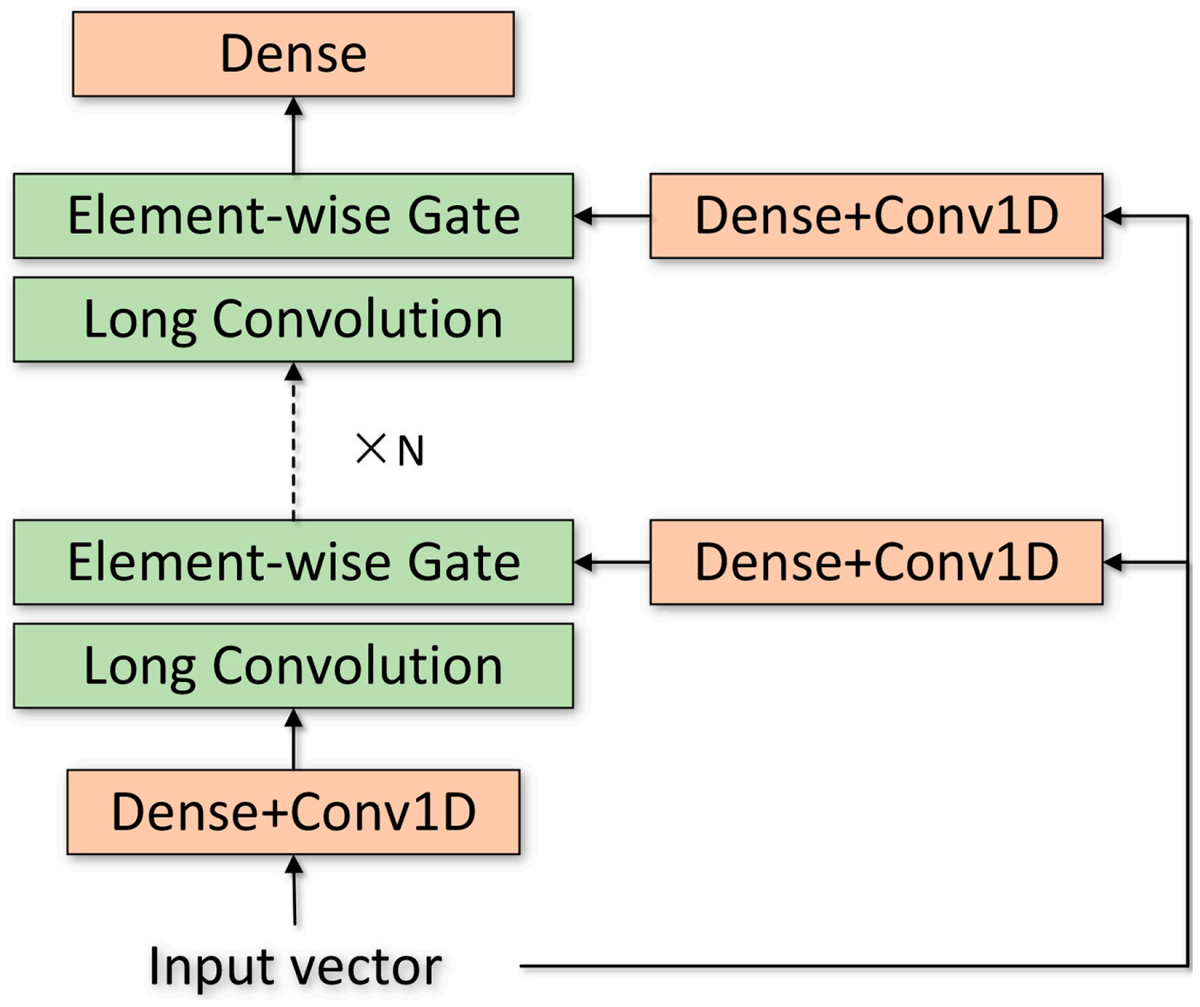
Illustration of Hyena operator, which is formed by the recurrence of long convolution and element-wise gating.

To capture both global and local information of protein sequences, we employ recurrent convolution and 1D ResNet architectures. Initially, the feature vectors of each protein are fed into a sub-quadratic replacement to the dense-attention mechanism, known as Hyena operator (Poli *et al.* 2023). As depicted in Fig. 2, Hyena operator interleaves long convolution with element-wise gating. In a Hyena operator with order *N*, there are N+1 linear projections for input vector *v*, denoted as $l^{i}$. The entire Hyena operator is defined as a recurrence of long convolution and linear projection:

(1) $$z^{i+1}=l^{i+1}(h^{i}*z^{i}),$$

where $z^{i}$ denotes the output of *i*th layer, $z^{1}=l^{1}$, and each $h^{i}$ denotes a learnable long convolution filter. In Hyena, $h^{i}$ contains a position encoding and an implicit convolution parameterized by an multilayer perceptron (MLP) and a Fast Fourier Transform convolution (fftconv). A dense layer is used after the final element-wise gate to match the output vector’s dimension with the input vector’s dimension.

A 1D ResNet is used for efficient learning of complex patterns in data. The 1D ResNet retains the standard ResNet architecture, substituting 2D convolution with 1D convolution to handle sequential data. It consists of skip connections, multiple convolution layers, and batch normalization. The skip connections in 1D ResNet help address the vanishing gradient problem when processing sequential data. The output of 1D ResNet is subsequently processed by a max pooling layer, a bidirectional gated recurrent unit (BiGRU), and a fully connected layer.

At this stage, the attention-free mechanism constructed by the Hyena operator, combined with the 1D ResNet architecture, enables the efficient extraction of long-range dependencies in protein sequences. In addition, the complexities of the Hyena operator and 1D ResNet are sub-quadratic and linear with respect to sequence length, respectively. Overall, this stage maintains efficient and effective extraction of contextualized information.

### 2.2 Anti-symmetric GCN for protein-relation representation

We incorporate anti-symmetric operation and graph convolution layer to learn the long-range relational information between proteins. Initially, the input PPI graph G=(P,X,Y) and protein vector representation are processed by GraphSAGE block, which focuses on inductive representation learning and exhibits better generalization ability for unseen proteins than the commonly used GIN block for PPI prediction.

A GraphSAGE block uniformly samples a fixed-size set of neighbors of protein *p*, denoted as U(p). The protein embedding of protein *p* is

(2) $$h_{p}=\text{ReLU}(W^{\left(1\right)}\cdot \text{CONCAT}(v_{p},h_{U(p)})),$$

where $W^{\left(1\right)}$ denotes the weight matrix and $v_{p}$ denotes the protein embedding of the previous block, for the first GraphSAGE block, *v* is the feature vector computed by contextualized information extraction stage, $h_{U(p)}=\text{AVERAGE}(\{v_{u},\forall u\in U(p)\})$. The GraphSAGE block contains a standard GepahSAGE layer, followed by ReLU activation and batch normalization.

To further refine the vector representations of proteins and mitigate over-smoothing caused by the imbalanced distribution of node degrees in the PPI dataset, we are inspired by the Anti-Symmetric Deep Graph Network (Gravina *et al.* 2022), which incorporates anti-symmetric operations in the update of node representation. The anti-symmetric GCN provides a forward Euler democratization of a stable ordinary differential equation on graph structure. Denoting the input vector representation of protein *p* as $v_{p}$, we iteratively compute the updated $h_{p}$ by the previous representation of *p* and its neighborhood as follows:

(3) $$h_{p}=v_{p}+\epsilon \sigma ((W^{\left(2\right)}-W^{(2)T}-\gamma I)v_{p}+\phi (V,N_{p})+b),$$

where $W^{(2)T}$ is the weight matrix of anti-symmetric GCN, *I* is the identity matrix, and γ is the hyper-parameter for diffusion strength regulation. The term $(W^{\left(2\right)}-W^{(2)T}-\gamma I)v_{p}$ formulates an anti-symmetric representation of $v_{p}$. This representation is added with bias vector *b* and aggregation of the neighborhood information of *p*. The sum vector is then processed with activation function σ and Euler discretization step ϵ subsequently. Finally, the output of discretization is added to input vector $v_{p}$. The function ϕ represents the standard graph convolution operation:

(4) $$\phi (W^{\left(3\right)},N_{p})=W^{\left(3\right)}\sum_{q\in N_{p}\cup p}\frac{1}{d_{q}d_{p}}v_{q},$$

where $N_{p}$ denotes the neighborhood set of protein *p*, $W^{\left(3\right)}$ denotes the weight matrix of graph convolution, $d_{p}$ and $d_{q}$ denote the degrees of the nodes corresponding to protein *p* and *q*. The anti-symmetric GCN block can be considered as a continuous process of diffusion on graph-structured data. The anti-symmetric weight matrix $\left(W^{\left(2\right)}-W^{(2)T}-\gamma I\right)$ imposes stability and conservative constraints, which enables both the generalization ability on nodes with different degrees and preservation of long-range relation between nodes.

### 2.3 Classifier and loss function

For protein pair $x_{ij}=\{p_{i},p_{j}\}$, the element-wise product operation is used to combine the feature vector of protein $p_{i}$ and $p_{j}$. A fully connected layer with Sigmoid activation is applied to the pair representation and used to compute the output for PPI employed as the classifier for PPI prediction, defined as ${\hat{y}}_{ij}=\text{Sigmoid}(\text{FC}(h_{p_{i}}\cdot h_{p_{j}}))$.

The entire learning architecture is trained to optimize asymmetric loss (Ridnik *et al.* 2021), which integrates cross entropy and asymmetric focusing into a unified formula:

(5) $$\begin{array}{l} \mathrm{ASL}=\{\begin{array}{c} L_{+}={\left(1-{\hat{y}}_{ij}\right)}^{\gamma }+\text{log}({\hat{y}}_{ij})\\ L_{-}={\left({\hat{y}}_{m}\right)}^{\gamma }-\text{log}(1-{\hat{y}}_{m}) \end{array} \end{array}$$

$$\begin{array}{c} {\hat{y}}_{m}=\text{max}(p-m,0),m\geq 0, \end{array}$$

where $L_{+}$ and $L_{-}$ denote the loss value for positive and negative samples of a specific type, respectively, ${\hat{y}}_{m}$ is the shifted probability and *m* is probability margin. The asymmetric loss allows applying types of asymmetry on positive and negative class, thereby reducing the influence of the majority class on the overall loss. The threshold is handled by focusing parameter γ and probability margin *m*. For training set $X_{\text{train}}$, $Y_{\text{train}}$ with *c* PPI types, the learnable parameters set Θ is trained by minimizing the asymmetric loss function:

(6) $$L(\Theta )=\sum_{k=1}^{c}\left(\sum_{x_{ij}\in X_{\text{train}}}-y_{ij}^{k}L_{+}-(1-y_{ij}^{k})L_{-}\right).$$

Compared to the cross entropy loss that commonly used for multi-task prediction, anti-symmetric loss replaces the original component of $\hat{y}$ with asymmetric focusing of the probability shifting of $\hat{y}$. As shown in Eq. (5), the shifted probability ${\hat{y}}_{m}$ balances the class within each type by discarding negatives samples with small predicted values, which also reduces the bias imported by other labels in multi-task prediction.

### 2.4 Feature computation

In the feature computation process, the protein sequences are transformed into numeric vectors to serve as the input for our proposed model BaPPI. As BaPPI contains the process for extracting contextualized information, the initial feature computation is based on residue representation, consisting of three components: the co-occurrence sequence similarity between amino acids, the one-hot encoding of amino acid physicochemical properties, and the position-specific scoring matrix (PSSM).

The residue representation of protein sequence is obtained by a pre-trained amino acid representation model (Yao *et al.* 2019), which is a word2vec-based model that is trained by 558 590 protein sequences from Swiss-Prot database. The physicochemical property is measured by the electrostatic and hydrophobic similarity between amino acids, the 20 amino acids are classified into eight types. PSSM represents the likelihood of each amino acid occurring at each position of the original protein sequence (Chen *et al.* 2020), the computation of PSSM is based on multiple sequence alignment. As the alignment process requires additional database and tools such as PSI-BLAST, the usage of PSSM features is optional for our model.

In BaPPI, the length of each protein sequence is standardized to a fixed value *L*. Shorter sequences are padded with whitespace, while longer sequences are truncated to the first *L* amino acids. We conducted a comparative experiment to assess the impact of *L* on the performance of BaPPI. The results of this experiment are presented in Table S2 of the Supplementary File. In practice, *L* is set as 512 to reach a balance between performance and computational efficiency.

## 3 Results and discussion

### 3.1 Experimental setting

#### 3.1.1 Benchmark datasets

In this study, we evaluate the performance of our model on the SHS27K and SHS148K datasets (Chen *et al.* 2019), which are benchmark datasets used in the previous works of other leading PPI prediction methods. SHS27K and SHS148K are human PPI sets derived from the STRING database (Szklarczyk *et al.* 2023), a database that contains critical assessments and interactions of PPIs. To comprehensively assess performance across different species, we also derive two benchmark datasets from *Saccharomyces cerevisiae* data in STRING, following the strategy of constructing SHS27K and SHS148K datasets. The two datasets are named SYS30K and SYS60K.

SHS27K contains 1690 protein sequences and 12 517 PPIs. SHS148K contains 5189 sequences and 44 488 PPIs. SYS30K contains 2685 sequences and 30 074 PPIs. SYS60K contains 3549 sequences and 60 357 PPIs. Detailed information about these benchmark datasets and the dataset construction strategy is available in Table S1 and Section 1 in the Supplementary File.

In previous studies, random partitioning was commonly used to divide the training and test sets for PPI prediction. However, random partition approach often results in a significant overlap between the proteins in the training and test sets, leading to overfitting and inflated performance metrics on the corresponding datasets (Chen *et al.* 2019, Kang *et al.* 2023). To reduce the number of proteins occurring in both the training and test sets, we employed breadth-first search (BFS) and depth-first search (DFS) algorithms (Lv *et al.* 2021, Kang *et al.* 2023, Rao *et al.* 2024) to split the datasets. These two schemes were proposed by Lv *et al.* (2021) for a more consistent assessment across datasets, the BFS partition selects closely interacting proteins first, while the DFS partition tends to select sparsely distributed proteins in the PPI network. In our procedure, both partition schemes selected 20% of the interactions from each dataset, using the selected interactions as the test set and the remaining interactions as the training set.

We conducted the experiment five times; the final result is the average and standard deviation of the experiments, reported to two decimal places.

#### 3.1.2 Baselines

We compare our method against four state-of-the-art methods for sequence-based PPI prediction, including Protein–Protein Interaction Prediction Based on Siamese Residual RCNN (PIPR) (Chen *et al.* 2019), GNN-PPI (Lv *et al.* 2021), multi-hop neural network (LDMGNN) (Zhong *et al.* 2022), and attention free transformer and graph attention network (AFTGAN) (Kang *et al.* 2023). PIPR is a general framework for multi-type PPI prediction, binary PPI prediction, and binding affinity estimation, while the other methods are specialized for multi-task PPI prediction.

To evaluate our model’s performance relative to general deep learning approaches, we also compare it with GCNs and deep neural networks (DNNs). Since GCN and DNN lack mechanisms to capture the sequential order in features, we select six nominal features as input, including the transition feature (Dubchak *et al.* 1995), amino acid composiiton (AAC) (Shepherd *et al.* 2003), Conjoint triad (Shen *et al.* 2007), 3-mer amino acid embedding (Li *et al.* 2021), global position information (Li *et al.* 2021), and pseudo-amino acid composition (PAAC) (Yang *et al.* 2023).

#### 3.1.3 Evaluation metrics

Following the evaluation criteria employed in prior studies, we utilize Micro-F1 as the primary metric for assessing performance. Micro-F1 is computed using the harmonic mean of precision and recall averages, rendering it suitable for the imbalanced nature of PPI datasets. Furthermore, a detailed case study that evaluates accuracy, precision, recall, Matthew’s correlation coefficient (MCC), Hamming Loss (HL), the area under the receiver operating characteristic curve (AUROC), and the area under the precision-recall curve (AUPRC) is conducted to provide a comprehensive analysis of PPI prediction methods. Additionally, we compare the node degree of top predictions to assess the model’s capability in managing the inherent imbalance in PPI datasets.

In all experiments, PSSM was used to enhance the flexibility of BaPPI. The input features consist only of co-occurrence sequence similarity between amino acids and one-hot encoding of physicochemical properties.

### 3.2 BaPPI shows the best performance and generalization ability

Table 1 presents the Micro-F1 results of BaPPI and leading baseline methods. BaPPI obtains the best performance across all datasets. AFTGAN ranks second in seven cases (SHS27K, SHS148K, and the DFS of SYS60K), while LDMGNN ranks second in the remaining five cases. On average, BaPPI surpasses AFTGAN by 5.9%, highlighting the superiority of our framework. In addition, BaPPI exhibits a lower standard deviation compared to other methods in most cases. Overall, our proposed model demonstrates remarkable generalization capability and stability in predicting PPIs.

**Table 1.** Micro-F1 (%) comparison of methods on SHS27K, SHS148K, SYS30K, and SYS60K.

Dataset	Partition scheme	Method						
		BaPPI	AFTGAN	LDMGNN	GNN-PPI	PIPR	GCN	DNN
SHS27K	BFS	**75.87 ± 0.86**	68.83 ± 0.75	67.21 ± 0.87	64.74 ± 0.66	48.18 ± 3.80	58.30 ± 2.03	53.41 ± 2.25
	DFS	**76.69 ± 0.65**	70.44 ± 0.83	68.12 ± 0.66	69.08 ± 0.97	54.57 ± 1.53	59.51 ± 0.21	52.78 ± 0.63
SHS148K	BFS	**79.46 ± 0.43**	71.19 ± 1.42	71.86 ± 1.02	66.72 ± 1.41	56.80 ± 1.42	57.62 ± 0.79	43.37 ± 1.27
	DFS	**83.30 ± 0.22**	80.97 ± 0.66	78.47 ± 0.35	71.27 ± 0.70	58.95 ± 1.26	64.04 ± 0.10	56.47 ± 1.25
SYS30K	BFS	**82.08 ± 0.48**	73.59 ± 2.14	75.91 ± 0.88	71.92 ± 2.25	63.80 ± 2.14	67.66 ± 0.75	60.27 ± 0.07
	DFS	**83.67 ± 0.25**	75.94 ± 1.21	78.55 ± 0.28	75.54 ± 1.14	66.74 ± 1.69	72.44 ± 0.18	68.84 ± 0.12
SYS60K	BFS	**84.58 ± 0.23**	78.47 ± 2.68	80.52 ± 0.35	76.22 ± 0.43	66.17 ± 1.67	77.72 ± 0.19	73.87 ± 1.26
	DFS	**86.14 ± 0.11**	82.39 ± 3.56	82.86 ± 0.26	81.26 ± 1.04	74.62 ± 0.42	76.75 ± 0.25	72.73 ± 0.31

Bold text indicates the best result.

The comparison between different datasets shows that all methods perform relatively poorly on SHS27K compared to the other datasets. This could be attributed to the number of unknown proteins (proteins that only occur in the test set). In SHS27K, both the number of proteins and the average degree of each protein are smaller than in the other datasets. In addition, the performance of methods in the BFS scheme is generally lower than in the DFS scheme. The main reason is that BFS tends to collect proteins that are densely linked in the test set, increasing the number of unknown proteins in test set. As depicted in Table 1, BaPPI achieved higher improvements in the BFS scheme of SHS27K (6.7% compared to an average of 6.3% of the second-best method), suggesting its superior generalization ability in predicting interactions between unknown targets. In addition, although the performance of all methods decreases when switching from the DFS scheme to BFS, BaPPI maintains a significantly lower decrease, verifying its stability under different conditions.

The results of various baseline methods suggest that GNN-based approaches, such as BaPPI, AFTGAN, LDMGNN, GNN-PPI, and GCN, are more effective in PPI prediction than other methods like PIPR and DNN. The primary reason is that GNNs are inherently suitable for the graph structure derived from PPI networks. Furthermore, their ability to aggregate and update neighborhood information enables them to capture long-range dependencies between proteins.

### 3.3 In-depth analysis

To verify the learning ability of our proposed model, we conduct an in-depth analysis on SHS27K, including the performance in terms of various evaluation indicators and the trade-off between precision and recall at different thresholds.

First, we compare our proposed model with the other four PPI prediction methods using general evaluation metrics. As shown in Table 2, BaPPI maintains the highest accuracy. The difference between accuracy and micro-F1 for BaPPI (4.87%) is significantly lower than that of previous methods (ranging from 9.31% to 21.17%), indicating that BaPPI effectively overcomes bias toward the majority class in PPI dataset. BaPPI significantly outperforms the other methods in terms of recall, although it shows a slight disadvantage in precision. Compared to AFTGAN, BaPPI achieves a 17.22% increase in recall with only a 0.090% decrease in precision, suggesting an effective trade-off between precision and recall. BaPPI also achieves a much higher MCC, verifying its balanced performance across all PPI types and its robustness to imbalance. Furthermore, our proposed model surpasses baseline models in both AUROC and AUPRC, demonstrating its threshold independence and ability to identify positive PPIs. The advantage of our proposed model in MCC, HL, and AUPRC verifies its performance across all PPI types and effectiveness in handling imbalanced datasets.

**Table 2.** The detailed comparison of PPI methods on BFS scheme in SHS27K, each result is the average of five experiments.

	BaPPI	AFTGAN	LDMGNN	GNN-PPI	PIPR
ACC (%)	**80.74**	78.14	78.62	75.50	69.35
Precision (%)	65.70	**66.60**	70.59	63.09	55.50
Recall (%)	**89.52**	71.30	65.17	64.49	43.03
MCC (%)	**62.46**	52.69	52.41	45.29	27.61
HL (%)	**19.26**	21.86	20.38	24.59	30.65
AUROC (%)	**90.59**	85.83	85.17	80.13	7247
AUPRC (%)	**81.53**	73.57	72.89	64.20	53.64

HL: Hamming Loss. Bold text indicates the best result.

**Figure 3.**
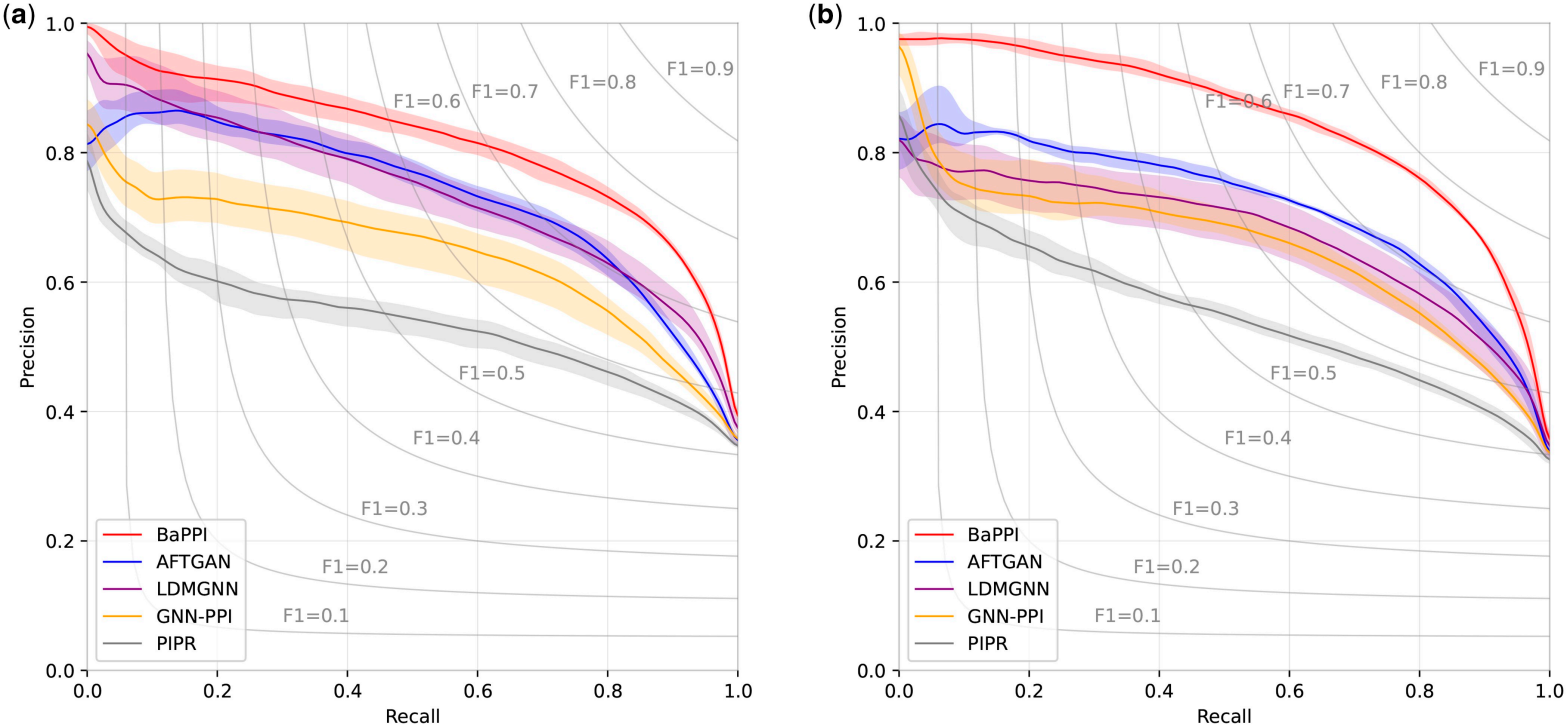
Precision–recall curves of PPI prediction of SHS27K, showing the performance of BaPPI compared to AFTGAN, LDMGNN, GNN-PPI, and PIPR. (a) Under the BFS partitioning and (b) under the DFS partitioning. The shade indicates the range between the highest and lowest results.

Second, we evaluate the threshold independence of each method by using precision–recall curves, as shown in Fig. 3. BaPPI obtains the best result in all thresholds, AFTGAN takes the second place in most of the thresholds. It is clear that the recall corresponding to best F1 is close to 0.8, with slight variations among different methods. For instance, the recall for the best F1 of GNN-PPI is around 0.77. Overall, the precision of BaPPI is much more stable than state-of-the-art methods as recall increases from 0 to 0.8, suggesting its strong threshold independence. This advantage is more pronounced in the DFS scheme compared to the BFS scheme.

### 3.4 Anti-symmetric operation and long convolution contribute to PPI prediction

To evaluate the contribution of different components in our model, we further conduct an ablation study. In the following scenario, w/o Anti-symmetric indicates the omission of anti-symmetric GCN and asymmetric loss function, while w/o long convolution (LC) refers to the exclusion of contextualized feature extraction of long convolution. The ablation study is performed on all benchmark datasets, and the *F*1-score is utilized to assess the prediction.

The results of ablation study are illustrated in Table 3. Our proposed model’s performance declines after the removal of anti-symmetric and long convolution, demonstrating that both components contribute to the enhancement of BaPPI. The performance reduction from removing long convolution is more pronounced than that from removing anti-symmetric components in SHS27K, SHS148K, and SYS30K, indicating that long convolution is more crucial in most cases. However, using only anti-symmetric or only long convolution is inferior to applying them simultaneously. The integration of these two components also reduces the variants in performance across most datasets.

**Table 3.** Ablation study on anti-symmetric structure and the long convolution, the result is micro-F1 (%) with two digits.

Dataset	Partition scheme	BaPPI	w/o Anti-symmetric	w/o LC
SHS27K	BFS	**75.87 ± 0.86**	72.00 ± 0.21	68.01 ± 0.89
	DFS	**76.69 ± 0.65**	72.83 ± 0.13	68.40 ± 0.95
SHS148K	BFS	**79.46 ± 0.43**	70.57 ± 0.13	70.26 ± 0.74
	DFS	**83.30 ± 0.22**	73.76 ± 0.72	77.25 ± 0.36
SYS30K	BFS	**82.08 ± 0.48**	77.53 ± 1.28	74.97 ± 0.59
	DFS	**83.67 ± 0.25**	79.34 ± 1.03	78.88 ± 0.62
SYS60K	BFS	**84.58 ± 0.23**	79.85 ± 1.49	83.15 ± 0.63
	DFS	**86.14 ± 0.11**	85.83 ± 0.65	85.91 ± 0.41

w/o LC: without the recurrent long convolution of Hyena Block.

Bold text indicates the best result.

### 3.5 Patterns of predicted PPIs

**Figure 4.**
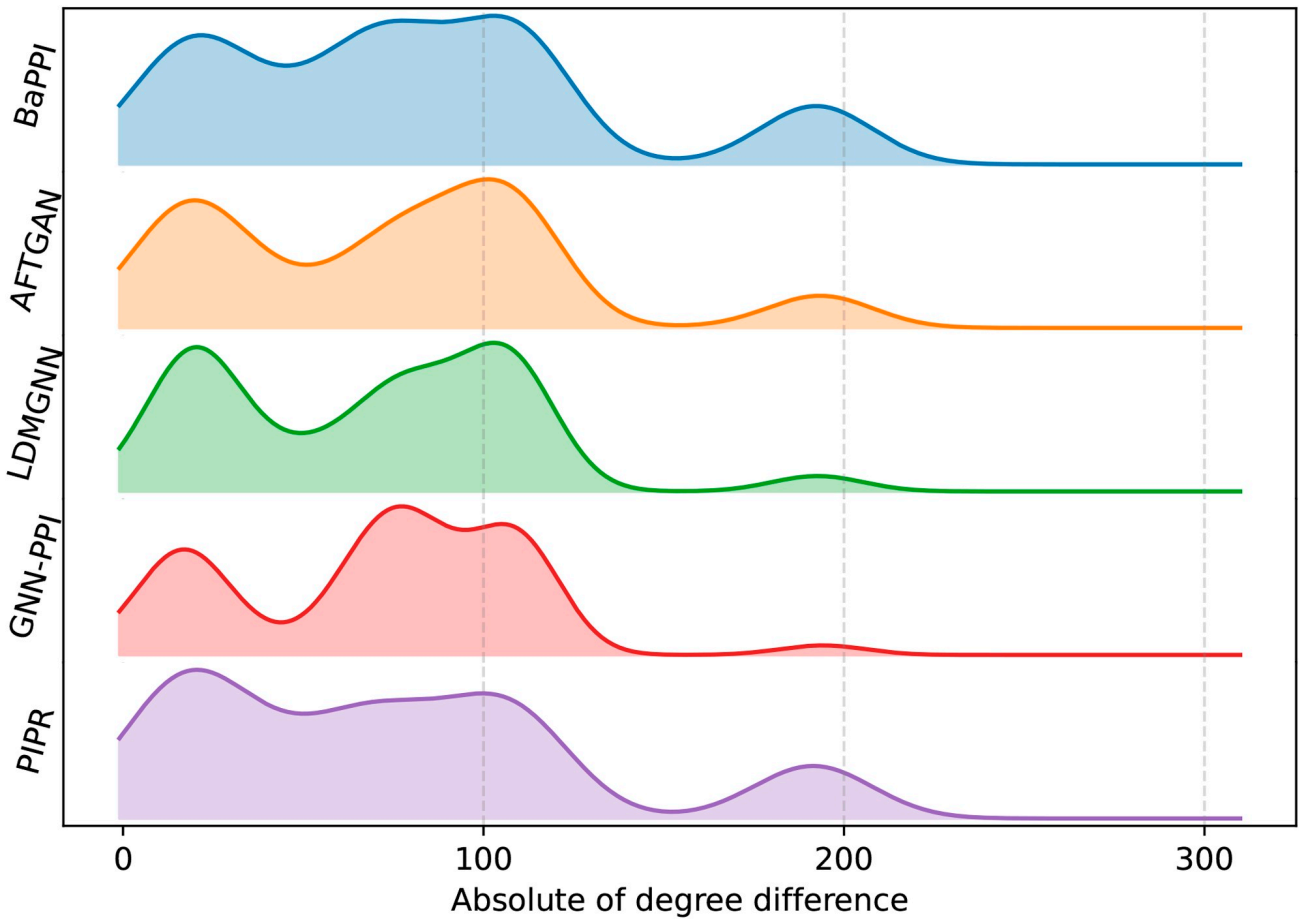
Patterns of the top 500 PPIs predicted by BaPPI and baseline methods in the BFS scheme of SHS27K. The *x*-axis represents the absolute value of the degree difference between the interacting proteins, while the *y*-axis indicates the percentage.

To assess the specific patterns among the PPIs predicted by each method, we selected the top 500 predictions from each method in SHS27K and computed the distribution of degree differences in each of these predictions, as shown in Fig. 4. Overall, our proposed model, BaPPI, exhibits significantly lower variance in prediction than other methods when the degree difference changes. Notably, although BaPPI is a GNN-based method, the variation caused by degree differences is much lower than that of other GNN-based methods. The variance of BaPPI is close to that of PIPR, a method not based on GNN. This indicates BaPPI’s higher resilience to variations in neighborhood information within the graph structure.

We observed some general patterns among different methods, such as relatively high proportions when the degree difference is around 25, 110, and 180. There is an increase when degree difference changes from 0 to 25 and a decrease when degree difference changes from 25 to 50. These patterns suggest that PPIs have a higher likelihood of occurring when the degree difference between corresponding proteins is approximately between 20 and 100.

Among the baseline methods, LDMGNN and GNN-PPI exhibit higher fluctuations when the degree difference ranges from 20 to 80. AFTGAN tends to predict PPIs when the degree difference is 105, while PIPR tends to predict PPIs with a degree difference of 20. Additionally, PIPR also predicts more PPIs when the degree difference is around 180.

### 3.6 BaPPI accurately identifies the minority PPI types

Typically, PPI types are unevenly distributed in PPI datasets. In the STRING database, a PPI can belong to one or more of the following categories: reaction, binding, post-translational modification (ptmod), activation, inhibition, catalysis, and expression. As shown in Table 4, the percentages of ptmod, activation, inhibition and expression are less than 5%, while each of the other three types accounts for more than 20%.

**Table 4.** The micro-F1 (%) of each type in SHS27K.

Type	Ratio	BaPPI	AFTGAN	LDMGNN	GNN-PPI	PIPR
Reaction	34.97	79.26	70.71	68.93	66.87	47.39
Binding	33.73	78.13	73.94	68.99	69.93	56.99
ptmod	1.85	70.69	61.31	56.32	47.85	30.82
Activation	4.86	71.79	63.31	66.02	63.69	56.30
Inhibition	3.09	69.11	62.63	61.55	44.60	31.06
Catalysis	20.91	84.85	80.38	81.64	76.42	49.22
Expression	0.60	63.57	34.91	48.61	27.18	12.90

The performance of each method on each type is illustrated in Table 4. BaPPI outperforms the other methods, surpassing AFTGAN by larger than 0.08 in reaction, ptmod, activation, and expression, thus achieving a more significant improvement in these four types, three of which are minority types. For expression type (the rarest type), BaPPI attains a micro-*F*1 score of 0.6357, making it the only method to achieve a result higher than 50%. Overall, BaPPI accurately identifies the minority PPI types that were mislabeled by previous methods.

**Figure 5.**
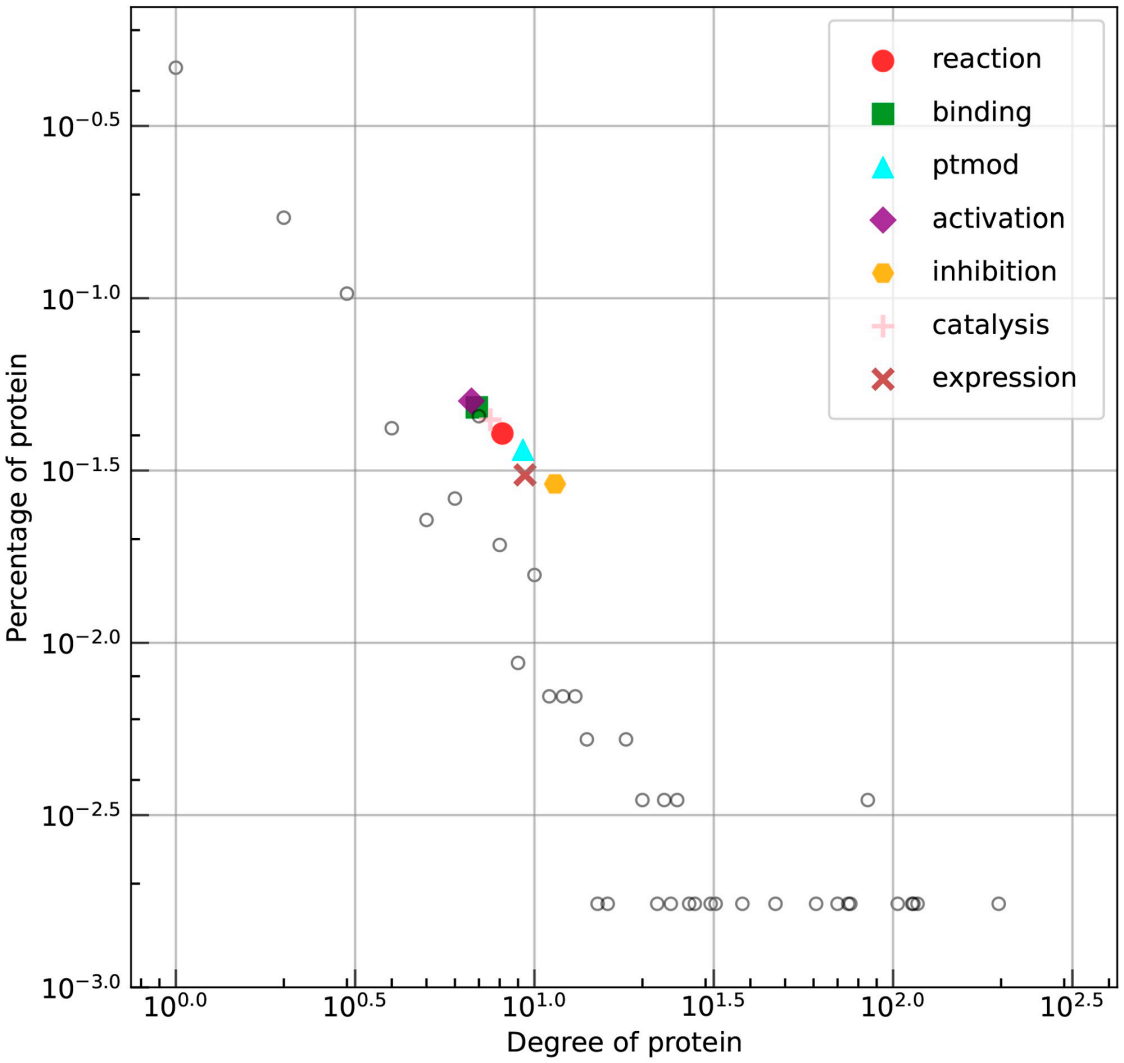
The degree distribution of proteins in SHS27K (unfilled circles) and the mean degree of the proteins involved in each type as predicted by BaPPI, depicted in a log–log plot.

To investigate the specific patterns within each type, we plot the degree distribution of proteins and the mean degree of proteins involved in the prediction of BaPPI, as shown in Fig. 5. It can be observed that inhibition, expression, and ptmod have the highest mean degree; the proteins involved with minority types typically have a higher degree than those associated with majority types. It should be noted that SHS27K filters sequences with more than 40% sequence identity and adjusts the percentage of each interaction type to create a more balanced dataset. As a result, its PPI type distribution differs from that of the original database.

## 4 Conclusion

In this paper, we propose BaPPI, a novel deep learning model based on anti-symmetric operation and long convolution for balanced prediction of PPI. By incorporating the asymmetric operation in GCN and cross-entropy, BaPPI effectively reduces the bias introduced by the imbalanced distribution of node degree and PPI types. The experimental results on four benchmark datasets show the promising performance of our framework on PPI prediction, supporting that BaPPI has strong generalizability and robustness to the inherent imbalance nature of PPI dataset. The improved predictive accuracy offered by BaPPI may significantly benefit future research and development in proteomics.

While BaPPI has demonstrated satisfactory prediction performance, there are several areas for future improvement: (i) exploring the PPI score included in the STRING database may contribute to a better understanding of prediction results, (ii) BaPPI is a sequence-based deep learning model, and incorporating structural data could improve PPI prediction quality, (iii) large-scale protein language models, such as evolutionary-scale models, could be explored for amino acid encoding, (iv) BaPPI’s memory usage scales quadratically with the average input sequence length, limiting its applicability to long protein sequences, so developing a more efficient feature extraction model would help address this limitation, (v) new numeric PPI characteristics available in the latest STRING database could be integrated to enhance the understanding of PPIs..

## Supplementary Material

btae603_Supplementary_Data
