# Supplementary material for: Anti-symmetric framework for balanced learning of protein–protein interactions
Source: Bioinformatics. 2024 Oct 15;40(10):btae603. doi: 10.1093/bioinformatics/btae603 (PMC11513017; doi:10.1093/bioinformatics/btae603)
Supplement: btae603_Supplementary_Data [file btae603_supplementary_data.pdf]

Supplementary file: Asymmetric-based framework for efficient balanced learning of protein–protein interaction

September 11, 2024

1 Benchmark datasets

Table S1: The details of benchmark datasets

	SHS27K	SHS148K	SYS30K	SYS60K
sequence number	$1,690$	$5,189$	$2,685$	$3,549$
interaction number	$12,517$	$44,488$	$30,074$	$60,357$
average degree	14.81	17.15	22.62	34.01
average sequence length	571	597	558	541
maximum sequence length	$5,171$	$5,636$	$4,910$	$4,901$
minimum sequence length	51	45	51	50

Table S2: The percentage (%) of PPI type in each dataset

	SHS27K	SHS148K	STRING human	SYS30K	SYS60K	STRING Yeast
reaction	18.22	17.71	25.46	21.91	21.44	18.64
binding	23.13	22.98	33.82	32.32	33.54	40.14
ptmod	7.5	9.15	3.45	6.39	6.22	5.75
activation	18.98	18.53	8.74	11.07	10.94	10.62
inhibition	8.1	8.81	3.28	3.4	3.54	3.26
catalysis	20.11	19.47	24.22	20.26	19.76	17.56
expression	3.96	3.35	1.03	4.65	4.56	4.03

2 Details of features for the GCN and DNN that used as baseline

Six features are used as input for Graph Convolutional Networks (GCN) and Deep Neural Networks (DNN). The details of each feature are as follows:

Conjoint triad: The conjoint triad feature combines k-mer and amino acid type. The k-mer denotes substrings of length k within a biological sequence, commonly used in proteomic and genomic data analysis. In the computation of conjoint triads, amino acids are divided into seven classes. With k set to 3, each 3-mer is considered a unit, and all 3-mers are classified based on the classes of their amino acids. Therefore, each protein sequence is represented by $7^3 = 343$ entries.

Amino acid composition (AAC): AAC calculates the fraction of each amino acid within a protein sequence. Each protein is represented as a vector with 20 entries, where each entry indicates the occurrence of one amino acid within the sequence, normalized by the sequence length.

Pseudo-amino acid composition (PAAC): A combination of conventional AAC and discrete sequence correlation factors, for sequence $S = [a_1, a_2, ..., a_L]$, the λ-order correlation factor is defined as:

$$\begin{aligned} \theta_\lambda &= \frac{1}{L-\lambda}\sum_{i=1}^{L-\lambda} O(a_i, a_{i+\lambda}) \\ O(a_i, a_j) &= \frac{1}{3}[(H_1(R_j) - H_1(R_i))^2 \\ &+ (H_2(R_j) - H_2(R_i))^2 \\ &+ (M(R_j) - M(R_i))^2] \end{aligned} \tag{1}$$

$H_1(a), H_2(a)$ and $M(a)$ denotes the hydrophobicity, hydrophilicity and side-chain mass of a. The sequence is represented as a vector with length $20 + \lambda$:

$$v_i = \begin{cases} \frac{f_i}{\sum_{j=1}^{20} f_j + \omega \sum\limits_{k=1}^{\lambda} \theta_k}, & i <= 20 \\ \frac{\omega \theta_{i-20}}{\sum_{j=1}^{20} f_j + \omega \sum_{k=1}^{\lambda} \theta_k}, & i > 20 \end{cases} \\ i \in 1, 2..., (20 + \lambda) \tag{2}$$

f_i denotes the frequency of ith amino acid in sequence. It is clear that λ should be smaller than L, in practice, Chou et al. set λ as 30, we follow the same strategy in this work.

CTDT: The transition descriptor in CTD (Composition, Transition and Distribution). For 13 physicochemical properties of amino acids, the amino acids are divided into three functional groups by each property, and transition (T) is the frequency of dipeptides. Finally, a vector with length 39 is computed by the original sequence.

ProVec1D: A feature derived from ProtVec, in which each 3-mer in protein sequence is mapped to a vector with length 100, ProVec1D sums the 100 components as single numeric value.

Global position information: the sum of position information of each amino acid in protein divided by sequence length, where the position information denotes the relative position of amino acid in corresponding sequence.

3 The comparison of BaPPI with different trimmed length

Table S3: Micro-F1 (%) comparison of BaPPI with different trimmed length on SHS27K, SHS148K, SYS30K and SYS60K

Dataset	Partition scheme	Trimmed Length			
		128	256	512	1024
SHS27K	BFS	75.28 ± 0.78	75.88 ± 0.64	75.87 ± 0.86	$\mathbf{76.30 \pm 0.72}$
	DFS	76.29 ± 0.82	76.30 ± 0.32	$\mathbf{76.69 \pm 0.65}$	75.95 ± 0.33
SHS148K	BFS	78.92 ± 0.51	79.01 ± 0.32	79.46 ± 0.43	$\mathbf{79.65 \pm 0.33}$
	DFS	82.62 ± 0.38	83.13 ± 0.36	$\mathbf{83.30 \pm 0.22}$	83.07 ± 0.25
SYS30K	BFS	81.77 ± 0.58	82.12 ± 0.36	$\mathbf{82.08 \pm 0.48}$	81.70 ± 0.49
	DFS	83.11 ± 0.39	83.16 ± 0.22	$\mathbf{83.67 \pm 0.25}$	83.25 ± 0.27
SYS60K	BFS	84.15 ± 0.21	$\mathbf{84.60 \pm 0.18}$	84.58 ± 0.23	83.84 ± 0.44
	DFS	85.76 ± 0.10	85.78 ± 0.18	86.14 ± 0.11	$\mathbf{86.31 \pm 0.21}$

Bold text indicates the best result.

4 Complexity analysis

The computational cost of a method is a crucial factor in evaluating its overall effectiveness. Here, we evaluate the communication and computational overheads of BaPPI. As shown in Table S3, BaPPI's running time is consistently under 60 minutes across all datasets, demonstrating its suitability for large-scale datasets. When the input size doubles, the running time increases to approximately 1.5 times, confirming a sub-quadratic computational complexity with respect to protein sequence length.

Table S4: Running time (in minute) of BaPPI with different trimmed length under the BFS scheme

Trimmed Length	128	256	512	1024
SHS27K	3.6	5.1	7.2	10.4
SHS148K	15.6	22.0	28.4	43.0
SHS30K	5.2	9.7	13.3	16.5
SHS60K	18.3	25.4	34.2	51.0

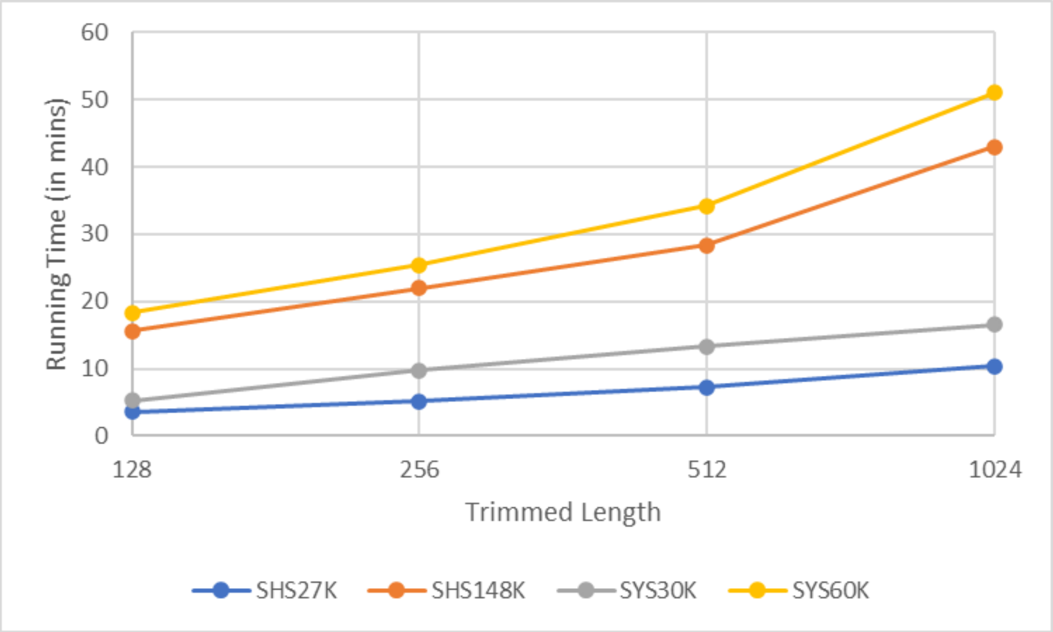

Figure S1: The running time of BaPPI with different trimmed length

5 Manual for BaPPI

The usage of BaPPI including the following steps:

1. Download all files from https://github.com/ttan6729/BaPPI
2. Check and implement the environment requirement that presented in above link.
3. BaPPI requires the following files as input: A sequence file that in csv or tsv format, each line contains the sequence name and amino acid sequence. A interaction file that in tsv format, each line contains protein A, protein B and one interaction type between them. The example files can be found in data folder.
4. Use `python3 main.py -h` to get all help message. An example command for running BaPPI is as following: `python3 main.py -m bfs -i data/SHS27K.txt -L 512 -o SHS27K-bfs -ln 3 -e 100`.
